# Development, results and prospects of the spring durum wheat breeding in Russia (post-Soviet states)

**DOI:** 10.18699/VJGB-23-71

**Published:** 2023-10

**Authors:** P.N. Malchikov, M.G. Myasnikova

**Affiliations:** Samara Federal Research Scientific Center of the Russian Academy of Sciences, Samara Scientific Research Agriculture Institute named after N.M. Tulajkov, Bezenchuk, Samara region, Russia Institute of Cytology and Genetics of the Siberian Branch of the Russian Academy of Sciences, Novosibirsk, Russia; Samara Federal Research Scientific Center of the Russian Academy of Sciences, Samara Scientific Research Agriculture Institute named after N.M. Tulajkov, Bezenchuk, Samara region, Russia

**Keywords:** durum wheat, Eurasian region, variety, breeding history, breeding rates, crop resistance, pathogen, yield, quality, protein, gluten, pigments, markers, твердая пшеница, Евразийский регион, сорт, история селекции, темпы селекции, устойчивость, патоген, урожайность, качество, белок, клейковина, пигменты, маркеры

## Abstract

The article outlines a brief historical background on the introduction to cultivation, distribution and breeding of spring durum wheat in the steppe and forest-steppe regions of Eurasia (the countries of the former USSR: Russia, Ukraine, and Kazakhstan). The approaches and methodology for improving durum wheat during certain scientific selection periods are given. The features of the selection program implementation and the breeding scale expansion during the creation of breeding stations at the beginning of the XX century, after the end of the Great Patriotic War, in the second half of the XX century, and at present are considered. A characteristic according to the main features and properties of varieties created in different periods is given. The achievements of the classical breeding method by comparing old and new varieties are analyzed. The efficiency and rate of wheat selection by periods in different regions of Russia is estimated. The results and methods of breeding for yield, resistance to drought, leaf diseases (Stagonospora nodorum Berk., Septoria tritici (Roeb. et Desm.), Bipolaris sorokiniana (Sacc.) Shoemaker, Pyrenophora tritici repentis (Died.) Drechs., Fusarium sp., Puccinia titicina Eriks., Puccinia graminis Pers. f. sp. tritici Eriks., Blumeria graminis (DC.) f. sp. tritici Em. Marchal), grain pathogens Ustilago tritici (Pers.) Rostr.) and pathogens causing darkening of the corcule and endosperm (Bipolaris sorokiniana (Sacc.) Shoemaker, Alternaria tenuis (Nees et Fr.), Аlternaria triticina (Prasada & Prabhu)), pests (Cephus pygmeus Lens, Osinosoma frit L., Mayetiola destructor (Say)), grain quality (protein content, amount of yellow pigments, dough rheology, sprouting resistance) and end products are presented. The prospects for the molecular marker application for a number of traits in breeding in the near future are given.

## Abstract (ru)

В статье представлен исторический обзор введения в культуру, распространения и селекции яровой твердой пшеницы в степных и лесостепных регионах Евразии (страны бывшего СССР – Россия, Украина, Казахстан). Приведены подходы и методология улучшения твердой пшеницы по периодам научной селекции. Рассматриваются особенности реализации программ и расширения масштабов селекции во время организации селекционных станций в начале ХХ в., после завершения Великой Отечественной войны, во второй половине ХХ в. и в настоящее время. Представлена характеристика по основным признакам и свойствам созданных в разные периоды сортов. Анализируются достижения классической селекционной методики путем сравнения старых и новых сортов. Дана оценка эффективности и темпов селекции в разных регионах России. Приведены результаты и методы селекции на урожайность, устойчивость к засухе, болезням листьев (Stagonospora nodorum Berk., Septoria tritici (Roeb. et Desm.), Bipolaris sorokiniana (Sacc.) Shoemaker, Pyrenophora tritici repentis (Died.) Drechs., Fusarium sp., Puccinia titicina Eriks., Puccinia graminis Pers. f. sp. tritici Eriks., Blumeria graminis (DC.) f. sp. tritici Em. Marchal.), патогенам зерна Ustilago tritici (Pers.) Rostr.) и патогенам, вызывающим потемнение зародыша и эндосперма (B. sorokiniana (Sacc.) Shoemaker, Alternaria tenuis (Nees et Fr.), Аlternaria triticina (Prasada& Prabhu)), вредителям (Cephus pygmeus Lens, Osinosoma frit L., Mayetiola destructor (Say)), по качеству зерна (содержание белка, желтых пигментов, реологические свойства теста, устойчивость к прорастанию) и конечных продуктов. Оцениваются перспективы применения в ближайшем будущем молекулярных маркеров в селекции по ряду признаков

## Introduction

**Fig. 1.**
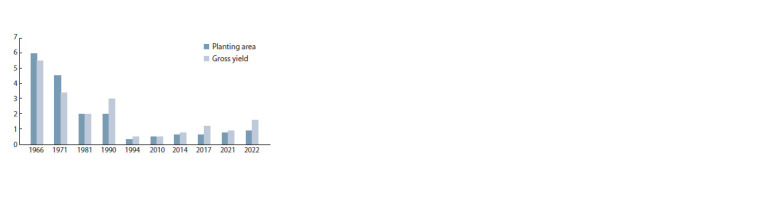
Dynamics of planting area (million ha) and gross grain yield (million tons) of durum wheat in Russia, 1966–2022.

Durum wheat is a crop of great importance in the production and consumption of specific food products (pasta, cereals, bread) that have a long shelf life, high nutritional value, and are in demand in almost all regions of the world. The main producers of durum wheat are (in million tons): the European Union – about 9.0 (including Italy – 4.3; France – 1.9; Greece – 1.1; Spain – 1.0); Canada – 5.2; Turkey – 3.7; the USA – 2.3; Kazakhstan, Syria, Algeria – 2.2 each; Morocco – 1.8; Mexico – 1.5; and Tunisia – 1.0 (Eurostat, 2019). In Russia, the domestic consumption of durum wheat grain is about 1.0 million tons per year, 0.2 t of which is imported mainly from Kazakhstan (Groshev, 2019). A significant reduction in planting area (from 2.0 million ha in 1990 to 0.45 million ha in 1994) and in production of durum wheat in Russia took place during the transition from a planned economy to a market economy in the 1990s (Fig. 1).

The durum wheat production decline can be explained by the higher competitiveness of winter crops (winter soft wheat, winter rye) and spring crops (barley, soft spring wheat) in relation to durum wheat which is represented mainly by spring cultivars in Russia. Durum wheat is grown without irrigation in a sharply continental arid climate with an annual rainfall of 250 to 450 mm per year. Biotic stress factors include pests such as aphids, bedbugs, thrips, grass flies, bread sawfly and pathogens such as Helminthosporium and Fusarium leaf spot, Septoria, powdery mildew, stem and in some years brown rust. The effect of pathogen harmfulness in epiphytotic years can be compared with the level of crop losses from severe drought (Vasilchuk, 2001; Rsaliev et, 2020; Tajibayev et al., 2021). The main challenges in spring durum wheat breeding in Russia are associated with the searching and implementation of opportunities to reduce the effect of these limiting environmental factors and improving the quality of grain and end products.

## Prehistory and early history of the durum wheat crop

On the former USSR territory, tetraploid wheat in the form of emmer T. dicoccum Schrank ex Schübler was cultivated in the areas of modern regions of Ukraine (Khmelnitsky region) and Azerbaijan in the IV millennium and in the II millennium BC, respectively. The appearance of durum wheat itself (T. durum Desf.) was noted in the IX century in Sumy oblast of modern Ukraine (Golik V.S., Golik O.V., 2008). Then, since the XVI–XVIII centuries, it has been widely distributed in the steppe and forest-steppe Russian regions – the central black soil region, the Volga region, the North Caucasus, the Urals, Western Siberia, Ukraine and Western Kazakhstan (Golik V.S., Golik O.V., 2008; Goncharov, 2012). Local cultivars – populations (landraces) under the names of Beloturka, Kubanka, Garnovka, Arnautka, etc. have been cultivated there.

For several centuries, as new territories were being developed, the area of cultivation of landraces increased, regional biotypes were formed simultaneously, which retained (at the time of introduction) the original names (for example, Kubanka), but in the process of long-term reproduction with a change in the dominant variety in the population, acquired regional features. Therefore, in the collection of Russian genetic resources of the N.I. Vavilov All-Russian Institute of Plant Genetic Resources (VIR), there are several cultivars Kubanka, Beloturka, etc., with a prefix to the name of the region of origin.

## Scientific breeding: development of a network of breeding institutions, general methodology and breeding results from the beginning of the XX century till present

Archival documents of Russian Department of Agriculture (Germantsev, Ilyina, 2019) contain a report on seed production dated 1848, which provides evidence that the peasants of Saratov Trans-Volga region (Novouzensky district) selected the best ears for seeding, i. e. improving mass breeding was carried out. The grain vitreousness and the high protein content in it were the main advantages of Russian durum wheat at that time. At the end of the XIX century, the Italian and French pasta industry worked Fig. 1. Dynamics of planting area (million ha) and gross grain yield exclusively on Russian durum wheat (Shekhurdin, 1961).

Scientific approaches to farming and plant improvement have been developed in Russian experimental institutions since the opening of Gorygoretsky Institute of Agriculture with experimental fields in 1840, as well as Riga Experimental Station in 1864, and Petrovsky Agricultural Academy in 1867. By 1917, about 400 experimental institutions including 44 experimental stations were operating in Russia. The study of durum wheat was carried out on experimental stations of the steppe regions of Ukraine, the Volga region, the South Urals, Siberia and Kazakhstan. In 1909, the scientific selection of this crop began at the Krasnokutsk breeding station in Saratov Trans- Volga region that was the centre of production of the most high-quality and demanded durum wheat at that time. Then, selection began at Saratov (1911) and Bezenchuk (1912) experimental stations, also located in the Volga region. At the same time, V.V. Talanov began durum wheat breeding at Yekaterinoslav Experimental Station (Ukraine) and continued it at West Siberian Experimental Station.

One of the main tasks assigned, in particular, to Krasnokutskaya breeding station by the Novouzensky district, was the breeding of more productive cultivars (Germantsev, Ilyina, 2019). There was no problem with the quality of grain grown in the Volga region, since any quantity of it was consumed by the markets of Russia and Europe (Chekhovich, 1924). Breeding work at Krasnokutsk station was led by K.Yu. Chekhovich, who later continued it at Bezenchuk Experimental Station. Under his supervision, the collection and study of durum wheat local samples from Samara, Saratov, Orenburg, Kuban and Ural regions were organized

The first cultivars in the state variety testing system in 1924 (State Variety Testing Network under the People’s Commissariat of Agriculture of the RSFSR) were: Kubanka 5, Gordeiforme 10, Gordeiforme 432, Gordeiforme 189, Melyanopus 69, Gordeiforme 111, Melyanopus 209, Gordeiforme of mass selection by A.I. Nosatovsky, Arnautka of Kochin, Mindum (selection from the Arnautka by Minnesota Experimental Station, the USA), whose originators were eight breeding institutions (Talanov, 1926). In the period from the late 1920s to the mid-1930s, Melyanopus 69, Gordeiforme 189, Gordeiforme 10, Gordeiforme 432, Gordeiforme 27, were approved for use and were most widely used in the USSR (Talanov, 1926). Cultivars Melyanopus 69 and Gordeiforme 189 occupied 2.7 and 0.35 million ha of crops in the USSR, respectively. The third most widespread cultivar was Gordeiforme 10 – 0.25 million ha. Melyanopus 69 and Gordeiforme 189 were created by breeding from local cultivars of the Novouzensky district of Samara province and Ural region, respectively. According to V.V. Talanov (1926), the selection was carried out by K.Yu. Chekhovich in 1911. After many years of research, under the supervision of academician P.N. Konstantinov, they were recommended for farm use.

**Fig. 2.**
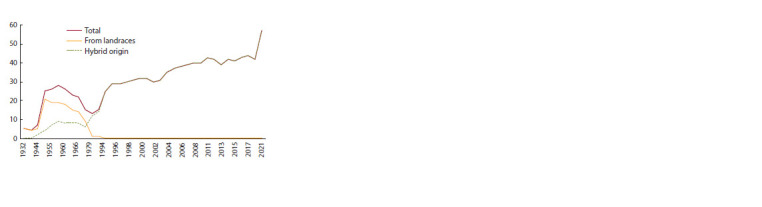
Dynamics of the number of durum wheat cultivars in the USSR (1932–1991) and in Russia (1992–2021), including those obtained by selection from landraces and hybridization method.

The morphological and botanical diversity of durum wheat local varieties required an explanation. According to K.Yu. Chekhovich (1924), it was necessary to solve a number of issues in physiology in order for the breeding to have a systematic character. The most successful work was carried out by methods of analytical selection on the source material of local varieties. Despite its effectiveness, collections of cultivars from different ecological and geographical groups were formed in all breeding institutions which were included in intraspecific and interspecific crosses. The breeding efficiency increased after the replenishment of genetic resources due to the expeditions of N.I. Vavilov, his discovery of the centres of origin of cultivated plants, the justification of a systematic approach to the search and study of the source material. However, breeding with the use of hybridization method has been more successful with soft wheat. Hybrid cultivars began to dominate among commercial soft wheat cultivars in the mid-1930s, among durum cultivars – by the early 1970s (Fig. 2).

This was because of the desire of government authorities and economic entities to obtain a high result in terms of yield and gross grain harvest that was more successfully achieved when cultivating soft wheat and concentrated the attention of breeders on it. The dominance in the USSR of the theory of T.D. Lysenko also reduced the efficiency of breeding including durum wheat in the 1950s. In the years of war 1941–1945, many breeding institutions in the occupied regions either stopped their activities or were evacuated, while part of the breeding material was lost (Golik V.S., Golik O.V., 2008). Institutions located in the rear, because of lack of funds and personnel, reduced research and breeding work. Nevertheless, in 1954, 25 cultivars were included in the catalog of commercial cultivars of spring durum wheat of the USSR (Catalogs…, 1954–1992). Selections from landraces (21 cultivars) dominated among them. The most widespread, as well as in the 1930s, were the cultivars of the Krasnokutskaya Breeding Station – Melyanopus 1932, Melyanopus 69, Gordeiforme 189, when Melyanopus 1932 was created from crossing landraces, Melyanopus 26 – from crossing Melyanopus 69/Melyanopus 1932.

For 40 years, cultivars of Krasnokutskaya Breeding Station held a monopoly, occupying in some years 86.0 % of durum wheat crops in the USSR (Germantsev, Ilyina, 2019). Melanopus 69 had been the standard of grain quality on the world market for almost 30 years (Shekhurdin, 1961; Dragavtsev et al., 1994). Cultivars Gordeiforme 432, Gordeiforme 5695 (Institute of the South-East, Saratov), Leukurum 33 (Bezenchuk Experimental Station) were widely distributed.

In Kazakhstan, cultivars of local breeding Akmolinka 5 with high quality and resistance to pathogens that cause black germ (Dorofeev et al., 1987) and Kustanaiskaya 14 were released. In the North Caucasus, the cultivar Krasnodarskaya 362 (Krasnodar Experimental Station) resistant to Swedish fly was introduced, obtained from crossing Gordeiforme 10 with a sample from Algeria. In Ukraine, the cultivar Narodnaya – selection from a local variety at the Kharkov Breeding Station had a significant distribution (0.9 million ha) in the 1950–1960s.

In the mid-1960s, 23 durum wheat cultivars were released in the USSR (Catalogs…, 1954–1992), among which 15 wear the selections from landraces while 8 were the selections from hybrid populations. The main areas of commercial crops were occupied by varieties Melyanopus 1932 and Melyanopus 26 of the Krasnokutskaya Breeding Station. In 1957, Kharkovskaya 46 cultivar was released, created by the Ukrainian Research Institute of Plant Breeding and Genetics from crossing line 34-5129 (interspecific hybrid T. turgidum/T. dicoccum) presumably either with Algerian durum wheat from the All-Union Research Institute of Plant Breeding collection, or with the line of Kharkov Station. Both versions could not be confirmed because of the loss of documentation during the Great Patriotic War (1941–1945) (Golik V.S., Golik O.V., 2008). That cultivar was distinguished by productivity, responsiveness to favourable environmental conditions, quite resistant to drought, and it was stood out in terms of protein and gluten content. By 1969, it occupied the main crop areas under durum wheat – 4.9 million ha in the USSR (Golik V.S., Golik O.V., 2008).

In the Middle Volga region, cultivars of the Kuibyshev (Bezenchukskaya) Breeding Station were released – Bezenchukskaya 102 (1962) and Bezenchukskaya 105 (1965). In the breeding record of both varieties there was the line Leukurum B-40, obtained by crossing Melyanopus 212 and Gordeiforme 1717 – an interspecific hybrid of landraces of soft and durum wheat. The cultivar Bezenchukskaya 105 (Blagonadezhdina, 1968) had the greatest practical value. It showed good combinational ability, crossings with Kharkovskaya 46 were especially promising, as they gave transgressions in terms of adaptability in the region of the Middle Volga and the Urals (cultivars Orenburgskaya 2, Orenburgskaya 10, Bezenchukskaya 182, Bezenchuksky yantar and their descendants) with great frequency.

In 1962, the cultivar of the Krasnoyarsk Research Institute of Agriculture – Raketa, obtained from interspecific hybridization – Gordeiforme 27*2/Zabaikalskaya emmer wheat was released. That cultivar was a good component for hybridization. It is included in the breeding record of two commercial cultivars with a high content of yellow pigments in the grain – Saratovskaya zolotistaya and Svetlana (Vasilchuk, 2001; Malchikov, Myasnikova, 2020).

In the next decade (1969–1979), the number of released durum wheat varieties in the USSR decreased to 13 (Catalogs…, 1954–1992). Among them there were only two cultivars of non-hybrid origin, one of them was Narodnaya in Ukraine, and the other was local Shavpkha in Georgia. The reduction in the total number of cultivars can be explained by the high level of productivity and competitiveness of Kharkovskaya 46 in all the regions. Cultivars of local importance were: Zernogradskaya 39, Krasnodarskaya 362 and Melyanopus 7 in the North Caucasus; Krasnokutka 6, Melyanopus 26, Leukurum 43, Saratovskaya 40, Saratovskaya 41 in the Volga region; Narodnaya and Nakat in Ukraine.

The catalog of zoned cultivars of the USSR in 1985 included 15 cultivars of durum wheat, 5 of which were from 1979: Almaz (Siberian Research Institute of Agriculture), Altaika (Altai Research Institute of Agriculture and Breeding), Bezenchukskaya 139 (Kuibyshev Research Institute of Agriculture), Orenburgskaya 2 (Orenburg Research Institute of Agriculture), Kharkovskaya 3 (Ukrainian Research Institute of Crop Production, Breeding and Genetics). In the same period, the cultivar Narodnaya, the last one obtained by selection from landraces, lost its commercial significance. Bezenchukskaya 139 was the most popular cultivar; in the years of maximum distribution, it occupied 1.5 million ha. In Siberia, the cultivar Almaz which was distinguished by its high protein content and the quality of pasta, was grown on 0.25–0.35 million ha. The drought-resistant and productive cultivar Orenburgskaya 2 spread in the Ural region (Dolgalev, Tikhonov, 2005). Kharkovskaya 3 was cultivated on irrigation in the Lower Volga region.

In the next decade (1986–1995), 18 cultivars were created that was 62.1 % of the total number of cultivars approved for use in the Russian Federation. Their originators were 10 institutions – 8 from Russia, one each from Ukraine and Kazakhstan (Catalogs…, 1954–1992; State Registers of Selection Achievements…, 1993–2022). The cultivars most widely distributed in terms of the number of tolerance regions were: Bezenchukskaya 182 (Samara Scientific Research Institute of Agriculture) – 5 regions, Krasnokutka 10 (Krasnokutskaya Breeding Station) – 5, Kharkovskaya 23 (Ukrainian Research Institute of Crop Production, Breeding and Genetics) – 4, Svetlana (Research Institute of Agriculture of the Central Chernozem Region) – 4, Orenburgskaya 10 (Orenburg Research Institute of Agriculture) – 4, Saratovskaya zolotistaya (Research Institute of Agriculture of the South-East) – 3, Voronezhskaya 7 (Research Institute of Agriculture of the Central Chernozem Region) – 2, Omsky rubin (Siberian Research Institute of Agriculture) – 2 regions. Cultivars of a wide range include Bezenchukskaya 182, Orenburgskaya 10, Krasnokutka 10, Kharkovskaya 23, Svetlana, Saratovskaya zolotistaya. The first two cultivars are well adapted to the conditions of the Middle Volga region, the Urals and the Central Chernozem region. Krasnokutka 10 with a high accumulation of protein and gluten in the grain is highly drought-resistant in the Lower Volga region. Kharkovskaya 23 was widely used in the Central Chernozem region and the Urals.

During that period, in the Research Institute of Agriculture of the South-East under the supervision of N.S. Vasilchuk (2001) a program for breeding high-quality cultivars was developed, methods for assessing the yellowness index of semolina, pasta, assessing the rheological properties of dough using a mixograph, farinograph, SDS sedimentation and culinary properties of pasta were introduced and improved. In the same institution, the cultivar Saratovskaya zolotistaya was created which significantly exceeded all previous cultivars in the concentration of yellow pigments in the grain.

From 1996 to 2006, 18 new cultivars of spring durum wheat were approved for use in Russia (State Registers of Selection Achievements…, 1993–2022). These cultivars were created at the Siberian Research Institute of Agriculture – 4, Altai Research Institute of Agriculture – 3, Research Institute of Agriculture of the South-East – 3, Samara Scientific Research Institute of Agriculture – 2, Bashkir Research Institute of Agriculture – 1, North-Donetsk Experimental Station – 1, Krasnodar Research Institute of Agriculture – 1. Two cultivars, Bezenchukskaya stepnaya and Steppe 3, were approved for use in three regions. Despite the reduction in the durum wheat crop area, the Bezenchukskaya stepnaya variety was actively used in the country during this period, occupying 120, 000 ha in some years.

With the development of regional selection, specialization of cultivars in ecological and geographical zones began to appear (Rozova et al., 2017). The conditional border of biotype specialization in Russia was marked on the territory of the Ural region, where cultivars originating from all six large agroecological zones (the North Caucasus, the Central Chernozem Region, the Lower Volga Region, the Middle Volga Region, the Urals, and Siberia) are competitive to one degree or another. The number of cultivars with a high content of yellow pigments in grains and with high gluten quality has increased. During 2007–2016 in all regions of Russia, 22 new cultivars were approved for use, i. e. the breeding rates averaged 2.2 cultivars per year that significantly exceeded all previous periods of durum wheat breeding. In 2016, the State register (State Registers of Selection Achievements…, 1993–2022) increased the total number of cultivars of spring durum wheat to 42.

The process of increasing the number of cultivars used can be seen as a movement towards the creation and diversification of cultivar systems. This is confirmed by both the number of successful breeding institutions (11) and their geography – from Irkutsk to Krasnodar. Along with the traditionally tall cultivars, short-stemmed ones Bezenchukskaya 209 and Rusticano, and medium-sized ones Bezenchukskaya 210, Bezenchukskaya zolotistaya, Lilek, Omskaya yantarnaya were registered. In terms of the duration of the growing season, the differences between early-ripening biotypes (Krasnokutka 13, Nikolasha) and late-ripening ones (Omsky izumrud, Omsky corund) in terms of heading date amounted to 10–12 days when tested in the Middle Volga region.

The number of cultivars with a high content of yellow pigments in the grain (at the level of Saratovskaya zolotistaya) continued to increase. Cultivar Bezenchukskaya zolotistaya exceeded this level by 25.0 %, reaching values of 8.5–9.0 ppm (Malchikov, Myasnikova, 2020). New cultivars were created that consistently form high-quality gluten: Bezenchukskaya 209, Bezenchukskaya niva, Bezenchukskaya zolotistaya, Luch 25, Annushka, Krassar, Lilek, Nikolasha. Breeders of the Altai Research Institute of Agriculture have created a cultivar Solnechnaya 573 which combines two properties that are difficult to combine – high yield in the tolerance regions (Western and Eastern Siberia) and high protein content. In the European part of the country, seven cultivars (Bezenchukskaya 205, Bezenchukskaya 210, Bezenchukskaya niva, Bezenchukskaya zolotistaya, Donskaya elegiya, Marina, Melodiya Dona) have been created which are approved for use in the Ural region. In Siberia, cultivars of only local selection have been released.

Over the past six years (2017–2022), 20 new cultivars have been created (State Registers of Selection Achievements..., 1993–2022) – 3.3 per year, so breeding rates increased compared to the previous period. Among them, cultivars recommended for use in one region prevailed – 18 to 90.0 %, which is a continuation of the trend that was determined at the previous stages – a gradual increase in the share of cultivars of local importance and regional diversification of cultivar systems. The specialization of cultivar systems at this stage is confirmed by a sharp increase in the number of short-stemmed cultivars carrying the RhtB1b gene: Triada, SI ATLANT, SI NILO and Tessadur that are recommended for the Central Chernozem region, Bourbon and Nikola that are for the Ural region. These cultivar are resistant to lodging, have a high productivity potential (it is 8.96 t/ha for Triada, 8.98 t/ha – for SI NILO).

Bezenchukskaya krepost, Kremen, and Melyana have middle-sized stems. The first one is included in the register for the Middle Volga and the Ural regions; both of the latter cultivars are only for the Ural region. Bezenchukskaya krepost is resistant to powdery mildew, brown rust, hard smut and accumulates in the grain almost the same amount of yellow pigments as the Bezenchukskaya zolotistaya cultivar. Obtained by LLC “Agroliga – Center for Plant Breeding” from the basic genotype of Samara Scientific Research Institute of Agriculture (1469D-59) by backcrossing to a cultivar from the USA Kofa (a donor of high quality gluten) and selection using molecular markers cultivar Taganrog is resistant to leaf spots, powdery mildew, grain sawfly and distinguished by a high content of yellow pigments and gluten quality. Cultivar Shukshinka is zoned in the Urals, Eastern, and Western Siberia, cultivar Oasis – in Western and Eastern Siberia and cultivar Omsky coral – in Western Siberia.

Cultivars Oasis and Omsky coral are middle-late, tall, but resistant to lodging with a realized grain yield potential of 5.7–6.2 t/ha. The cultivar Shukshinka is middle-late, medium-sized, resistant to lodging, has good and excellent pasta qualities, the maximum yield is 7.28 t/ha. Two cultivars, Yasenka and Yarina, were approved for use in the North Caucasus region. They are resistant to drought and lodging, loose smut, and are distinguished by large grains and good quality pasta. They are quite competitive in the Volga region and the Urals

## Source material and methods for creating genetic variation

At the beginning of scientific breeding, local cultivars were the main source material. They consisted of mixtures of cultivars and biotypes. Their study was carried out in accordance with the intraspecific classification of cultivated plants of F.K. Körnicke (1873) and J. Percival (1921) based on well-distinguishable features of the ear and grain. Subsequently, N.I. Vavilov (1940) proposed an ecologicalgeographical principle that combines the classification into cultivars according to F.K. Körnicke (1873) with the division of the entire diversity into ecological-geographical groups. The adequacy of such a division of landraces and ancient hexaploid wheat varieties was confirmed by the results of their clustering based on SSRs and RAPDs markers (Mitrofanova, 2012).

Separation of Eurasian durum wheat cultivars (Russia, Ukraine, Kazakhstan) of the steppe and forest-steppe ecotypes from groups of durum wheat from the Mediterranean and the Middle East is confirmed by the spread of various groups of alleles of gliadin-coding loci among the landraces of these regions (Kudryavtsev et al., 2014). Selection from landraces that was used in all breeding institutions proved to be effective. At the beginning, interspecific crossings of durum wheat (T. durum Desf.) with soft wheat (T. aestivum L.) and emmer wheat (T. dicoccum Schuebl.) prevailed in hybridization, after that – intraspecific crossings with the involvement of initial material from different ecological and geographical groups and from the other countries.

Landraces Beloturka and Sivouska were included in the breeding records of 53 and 41 % of released cultivars, respectively, which indicates the evolutionary nature of breeding with improving genetic systems of adaptability. Derivatives obtained from crossing durum wheat with emmer wheat were of great importance. Variety Kharkovskaya 46 created as a result of interspecific hybridization T. dicoccum, T. turgidum, and T. durum was included in the breeding record of 85 % of the cultivars included in the Russian register in 2004 (Martynov et al., 2005). Cultivar Raketa obtained using the sample T. dicoccum from Trans-Baikal territory through cultivars Saratovskaya zolotistaya, Svetlana is currently included in the breeding record of 36.0 % of commercial cultivars in Russia. Sample k-46995 T. dicoccum (All-Union Research Institute of Plant Breeding) through the cultivar Pamyati Chekhovicha participated in the origin of seven cultivars included in the register over the past eight years (State Registers of Selection Achievements…, 1993–2022). At all stages of breeding, soft wheat was used for hybridization. At present, 32.7 % of commercial cultivars have soft wheat among their ancestors

Triticum timopheevii (Zhuk.) cultivar was involved in hybridization as a source of resistance to pathogens. In the register of cultivars protected for 2022, there were three cultivars carrying a translocation on the 6B chromosome from T. timopheevii, which provides resistance to powdery mildew (Malchikov et al., 2015). In the 1970s, the commercial variety Melanopus 7 was obtained in Krasnodar with the involvement of T. timopheevii.

Since the 1930s, along with interspecific and intraspecific crosses, foreign cultivars have been involved in the creation of hybrid populations. The cultivars WSMP-13 (USA), Leucurum 983 (Italy) were widely included in crosses. WSMP-13 cultivar through the breeding line of the Samara Scientific Research Institute of Agriculture Gordeiforme 740 is included in the breeding record of 12 modern cultivars. The Research Institute of Agriculture of the South-East and the Federal Scientific Centre for Grain named after P.P. Lukyanenko use the gene pool from the international centre ICARDA, the USA and Canada – the cultivars Annushka and Krassar were obtained using the American cultivar Medora, the genes of the American line AWll/Sbl 4 were used for the cultivar Lilek. Omsky corund, Omsky coral were obtained from crossing with genotypes: k-47117, T 1004 = POD 11/Yazi 1 (CIMMYT). When creating short-stemmed varieties Bezenchukskaya 209 and Triada, the donors of the RhtB1b gene were Coccorit 71 and Anser 10 (CIMMYT). At present, the cultivar Pamyati Chekhovicha carrying the plant height reduction gene from the cultivar Ahninga (CIMMYT) is widely used for hybridization. In Federal Rostov Agricultural Research Centre, chemical mutagenesis and hybridization with foreign cultivars (Wells, Wascana) are used to induce genetic variability. As a result, cultivars Novodonskaya, Volnodonskaya, Donskaya elegy were created.

The main method of induction of genetic variability in durum wheat in Russia and in the countries of the former USSR is hybridization within and between species. Some scientists assign a crucial role to hybridization and estimate its contribution to the effectiveness of the breeding process up to 60.0 % (Vedder, 1992). The approaches used in the selection of parental components for hybridization correspond to the three principles proposed by S. Boroevich (1984): cultivar, trait, gene

The principles of cultivar and trait in the domestic literature are usually not separated. In this case, cultivars selected for hybridization are characterized by the entire range of breeding traits (Vasilchuk, 2001; Evdokimov et al., 2022). At the same time, the stage of prebreeding selection, or the purposeful creation of intermediate forms for the stepwise hybridization, is singled out (Shekhurdin, 1961; Vasilchuk, 2001). This principle of parental component selection is widely used in breeding for quantitative traits, resistance to drought and high temperatures. Methods based on the gene principle are used for: backcrossing – transferring genes to a specific gene pool, accumulation – combining genes in one genotype that determine different traits, “pyramidization” – combining two or more genes that determine one trait. The gene principle is used in breeding for resistance to pathogens, culm length and completeness, enzyme activity that are signs closely linked to biochemical or DNA markers

## Genetic diversity of commercial cultivars

The process of increasing uniformity in varietal populations is undesirable, as it increases the likelihood of rapid development of epiphytoties, the spread of pests, and the vulnerability of the varieties to the effects of other extreme environmental factors over a large area (Jacques et al., 2014). Genealogical analysis of the varieties released on the territory of Russia in 1929–2004 based on relatedness coefficients showed an increase in genetic diversity. At the same time, genetic erosion of local material was recorded – the number of Russian original ancestors of modern cultivars decreased by 20 %. In general, during this period, the lower genetic diversity threshold in all regional breeding centres did not reach a critical level corresponding to the similarity of half-sibs (r-coefficient of relatedness varied from 0.18 to 0.23 for breeding centres; r-coefficient for half-sibs – 0.25) (Martynov et al., 2005). Unambiguous trends in the change in genetic diversity for alleles of gliadin-coding loci during four historical (in time) evolution stages of the varietal population of spring durum wheat were not found (see the Table).

**Table 1.**
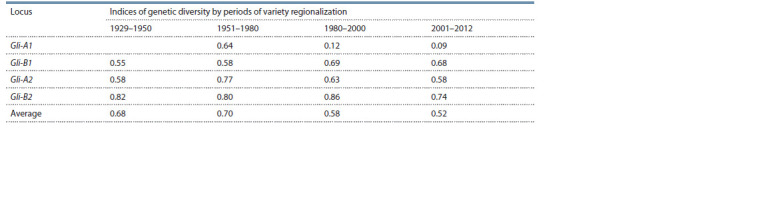
Nei’s indices of spring durum wheat genetic diversity from Russia and the former USSR for four gliadin-coding loci in a historical context (according to Kudryavtsev, et al., 2014)

The constancy of the allelic composition at the Gli-A1, Gli-B1, Gli-B2 loci was established for the first (1929– 1950) and second (1951–1980) stages. In cultivars of the second stage, the number of alleles at the Gli-A2 locus increased significantly that led to some increase in the coefficient of genetic diversity in general for 4 loci from 0.68 to 0.70. At the next stages, this trend was reversed. A significant narrowing of the diversity occurred at the third (Gli-A1, Gli-A2 loci) and especially the fourth stage, most strongly at the Gli-A1 locus. A significant decrease in the diversity coefficient at the fourth stage also occurred at the Gli-A2 and Gli-B2 loci, which was constant at throughout the XX century. Gli-B1 which is stable in composition of alleles includes allele c with a frequency of 0.46 which contains the electrophoretic component γ-45 that is a marker of genes determining the formation of high-quality gluten.

It is obvious that the current intensive selection of highquality cultivars can lead to a monoallelic state of the locus and a narrowing of the genetic diversity of the created cultivars. This negative trend can be overcome by attracting a genetically diverse source material, identifying new alleles and intensifying the variety creation in regional breeding centres (Kudryavtsev et al., 2014).

## Yield selection results

There is information in the scientific papers about the results of studying the yield trend in breeding institutions in the Volga region (Research Institute of Agriculture of the South-East, Samara Scientific Research Institute of Agriculture) and Western Siberia (Omsk Agricultural Research Centre, Federal Altai Scientific Centre for Agrobiotechnology). At the Samara Research Scientific Institute of Agriculture, the genetic component of yield has been determined quite accurately since the regular testing of Leukurum 33 cultivar in the 1930s. This cultivar of the second stage (Malchikov, Myasnikova, 2015), until the completion of testing the cultivar of the first stage – Gordeiforme 189 in 1958, exceeded it by 23.6 %. If we take the value of 5.0 % as the minimum difference between the yields of Gordeiforme 189 and landraces, then the breeding contribution to the increase in yield in the Middle Volga region when creating the Leukurum 33 cultivar is 28.5 % or 0.8 % per year.

The breeding contribution to the yield value during the creation of the cultivar Bezenchukskaya 105 (1965) and Kharkovskaya 46 (1968) increased by 5 %, but, at the same time, the growth rate due to the breeding decreased to 0.6 % per year from 1912, in the period from 1948 by 1965 – to 0.26 %. The main contribution to the increase in the yield of cultivars at the 2nd–3rd stage of breeding was associated with an improvement in the survival of plants at the time of maturation.

At the next stages – the creation of Bezenchukskaya 139 (stage 4, 1980), Bezenchukskaya 182 (stage 5, 1993), the genetic yield trend related to the variety of the 3rd stage – Kharkovskaya 46 was 10.1 %, related to the variety of the stage 4 – Bezenchukskaya 139 was 15.7 %, with breeding rates of 0.84 and 1.12 % per year, respectively (Malchikov, Myasnikova, 2015).

**Fig. 3.**
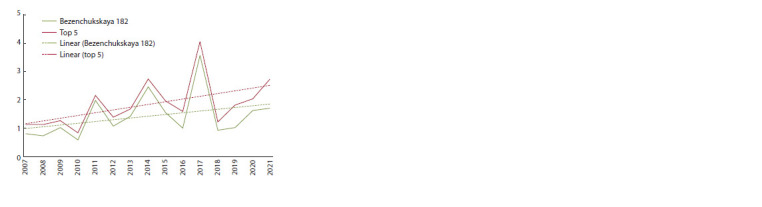
General and genetic trend (the ratio of the indicators of the five best breeding lines to the indicators of the cultivar of the 5th stage of breeding Bezenchukskaya 182) of durum wheat yield over the past 15 years (2007–2021).

Most often, the genotypic dispersion of the yield of cultivars of stages 3– 6 was associated with the variability of ear productivity traits and morphophysiological traits, such as Plant height, Harvest index – 65.5 and 31.1 % of cases, respectively. During the last 15 years (2007–2021), against the background of a general increase in the yield of durum wheat in competitive variety trials, an increase in selection improvement rate has been observed (Fig. 3). The trend in the increase in yield of Bezenchukskaya 182 variety from 1993 to 2021 was 28.5 % or 0.95 % per year.

Thus, over 110 years of durum wheat breeding at the Samara Scientific Research Institute of Agriculture, the genetic yield progress was 0.80 % per year or 87.7 % for the entire period, including the stages of breeding (varietal change): stages 1–2 – 28.5 %, stage 3 – 5 %, stage 4 – 10.0 %, stage 5 – 15.7 %, stage 6 – 28.5 %.

In the Research Institute of Agriculture of the South-East in the XX century (1929–1999), from the moment of the variety Gordeiforme 432 creation (selection from the local Beloturka), the genetic yield trend was 54 % (Vasilchuk, 2001). In the XXI century (2000–2017) it increased by 22 % and amounted to 73.0 % or 0.79 % per year in general for the entire breeding period (Gaponov et al., 2017). Breeding progress in terms of yield was associated primarily with an increase in grain size, the number of productive stems and Harvest index (HI). It was not possible to increase the number of spikelets per spike, the number of grains per spike and spikelet, with the exception of some cultivars. It is predicted that no cardinal changes in plant habitus (reduction in plant height) are expected (Vasilchuk, 2001; Gaponov et al., 2017).

The first cultivar of spring durum wheat created at the West Siberian Station (Omsk Agricultural Research Centre) was Gordeiforme 10 (Talanov, 1926). It was released from 1929 until 1960 and remained the main variety in the region. It was replaced by the Kharkovskaya 46 cultivar which surpassed Gordeiforme 10 by 10 % in the foreststeppe zone (Savitskaya et al., 1980). In 1979, the Almaz variety was released which exceeded Kharkovskaya 46 in yield by 0.23 t/ha or 9.7 % in Siberia and Kazakhstan. Over the next 20 years, Omsk rubin (1991), Angel (1997), and Omskaya yantarnaya (1999) were created. The yield trend for this period (from the Almaz cultivar to Omskaya yantarnaya) amounted to 0.6 t/ha or 22.5 %, i. e. 1.07 % per year based on the results of a long-term study at the Omsk Agricultural Research Centre (Evdokimov et al., 2021).

Cultivars of the last breeding period of durum wheat in the Omsk Agricultural Research Centre (2000–2021) – Omsky izumrud and Omsky coral in terms of grain yield exceeded Omskaya yantarnaya by 18.4 and 19.2 %, i. e. selection rates were about 0.9 % per year (Evdokimov et al., 2020). The general trend of yield improvement breeding if we count from the Gordeiforme 10 variety is 61 % or 0.67 % per year. The cultivar productivity increase in the process of breeding at the Omsk Agricultural Research Centre followed the path of improving the number of productive stems per 1 m2 and the number of grains per spike. The caryopsis weight changed insignificantly (Evdokimov et al., 2021).

In the Altai Research Institute of Agriculture (Federal Altai Scientific Centre for Agrobiotechnology), the selection of spring durum wheat began in 1929 after the organization of the Barnaul Experimental Station, but systematic and large-scale work has been carried out since 1970. For 50 years, 10 commercial cultivars have been created. The yield trend from Kharkovskaya 46, the main standard in the 1970s, to modern cultivars was 0.39–0.79 t/ha or 14–29 %, the breeding rate for yield was 0.38–0.58 % per year (Rozova et al., 2017). A general trend in the change in yield elements was not found with the exception of the tendency to increase the weight of the grain at all stages. At the same time, most cultivars of all breeding stages formed larger grains. Some varieties surpassed Kharkovskaya 46 in terms of the number of grains per ear and Harvest index.

Thus, the selection of spring durum wheat for yield in the main breeding centres of Russia is carried out quite effectively. The rate of its increase depending on the breeding centre is 0.58–0.80 % per year.

## Selection of cultivars resistant to pathogens and pests

At the beginning of breeding work with spring durum wheat in the USSR (Russia), much attention was paid to the creation of cultivars resistant to dust brand (Ustilago tritici (Pers.) Rostr.) that is explained by its distribution in the regions of cultivation, increased durum wheat species susceptibility to the pathogen and deterioration in the quality of products. In the 1920–1940s, Gordeiforme 10, Melyanopus 69, Raketa, Gordeiforme 27 were resistant against an infectious background (Shestakova, Vyushkov, 1975). On their basis, highly resistant varieties Bezenchukskaya 121, Bezenchukskaya 139, Svetlana, Valentina, Bezenchukskaya 205, Triada, etc. were obtained. Currently, among the cultivars included in the State Register of Russia there are about 40 % resistant ones which in combination with the use of systemic seed treaters, effectively restrains the development of this pathogen (Vyushkov, 2004).

The most harmful diseases parasitizing the Eurasian durum wheat are leaf spots: Stagonospora nodorum (Berk.), Septoria tritici (Roeb. et Desm.), Bipolaris sorokiniana (Sacc.), Pyrenophora tritici-repentis, Fusarium sp. (Koishibaev, 2018). Breeding for resistance to these pathogens is regional. There is no complete resistance or immunity in durum and soft wheat cultivars to leaf spots (Lamari et al., 1989). In a study by C. Chu et al. (2008), only 25 of 132 durum wheat accessions had high or partial resistance to P. tritici-repentis and S. nodorum (Berk.). A high level of partial resistance to P. tritici-repentis (Tan spot) and S. nodorum (Berk.) has been found in synthetic hexaploid wheat (SHW) and soft wheat (Xu et al., 2004; Singh P.K. et al., 2006). Sustainable sources of SHW and wild relatives can potentially be used to improve durum wheat (Singh P.K. et al., 2006).

In the system of the Kazakh-Siberian program (KASIB), the following samples of durum wheat were identified (highly resistant to leaf spot pathogens in the field): 1693d-71, 2021d-1, Gordeiforme 1591-21 (Samara Research Institute of Agriculture), D-2165 (Research Institute of Agriculture of the South-East), Gordeiforme 08-107-5, Gordeiforme 178-05-02, Gordeiforme 05-42-12 (Omsk Agricultural Research Centre) (Gultyaeva et al., 2020; Rsaliyev et al., 2020). A significant part of durum wheat cultivars included in the state register of Russia according to the description of the authors and the results of the study in the state variety network show resistance (R), medium resistance (MR) or medium susceptibility (MS) to these pathogens. Sufficiently effective selection for resistance is confirmed by the low frequency of occurrence of the dominant allele of the susceptibility gene to P. tritici-repentis – Tsn1. Among the 43 studied cultivars, it was identified only in two – Soyana and Gordeiforme 08-25-2 (Rsaliyev et al., 2020).

The cultivars of durum wheat from Russia and Kazakhstan are sufficiently resistant to leaf rust (P. triticina Eriks.) which confirms the thesis that resistance to this pathogen is higher in durum wheat than in soft wheat (Ordoñez, Kolmer, 2007). Most durum wheat varieties included in the Russian Register have field resistance to leaf rust. During the years of its epiphytoties, cultivars with this type of resistance reduce the yield, the weight of the grain and its fillness, but much less than the susceptible ones. It is advisable to have cultivars with field resistance in the steppe regions with a dry climate where the harmfulness of brown rust is low (Krupnov, 2016). For regions with an increased level of annual precipitation (> 450 mm), it is advisable to use immune cultivars (immunity type 0-1). Donors of resistance genes (immunity with type 0-1) are varieties of the Samara Research Institute of Agriculture Marina and Leukurum 1750. These lines were isolated according to the field assessment in the KASIB seed-plot against a natural infectious background in two ecopoints (Omsk, South Kazakhstan) – Kargala 223, Gordeiforme 178-05-2, Gordeiforme 05-42-12 and Triada.

The results of the multipathogenic test showed the absence of the Lr2a, Lr2b, Lr2c, Lr9, Lr15, Lr16, Lr17, Lr19, Lr20, Lr24, Lr26 genes in the Russian and Kazakh breeding material. The use of molecular markers did not reveal the genes Lr1, Lr3a, Lr9, Lr10, Lr19, Lr20, Lr24, Lr26, Lr34, Lr37 (Gultayeva et al., 2020). In Spain, they did not identify any known Lr-gene in the collection of durum wheat cultivars either (Martínez et al., 2007). Of all known leaf rust resistance genes, only Lr14, Lr23, Lr61 and Lr 79 are derived from durum wheat and spelt (McIntosh et al., 2013). In this regard, it is not clear whether durum wheat has the same resistance genes in the A and B genomes that are identified in soft wheat, or whether these genes are completely different (Gultayeva et al., 2020).

Currently, due to climate change, there is a danger of the spread and intensification of the harmfulness of stem rust (P. graminis f. sp. tritici) in the steppe regions of the Urals, the Volga region and Siberia (Gultyeva et al., 2020; Evdokimov et al., 2022). An analysis of the periodicity of the pathogen spread shows that durum and soft wheat are very vulnerable to outbreaks of stem rust in the world. The emergence and spread of the Ug99 race and its highly virulent strain TTKSK which overcomes most resistance genes, requires special attention of breeders, geneticists, and phytopathologists (Singh R.P. et al., 2015).

In Russia, cultivars of durum wheat are used that exhibit to some extent resistance to stem rust in the field. According to the State Commission for Testing and Protection of Breeding Achievements, out of 63 cultivars included in the Russian register 14 cultivars showed resistance from medium (MR) to high (R) against a natural infectious background. Highly resistant cultivars include commercial cultivars: Triada, Lilek, Omskaya stepnaya, Omsky coral, Omsky izumrud. The following were moderately stable: Nikolasha, Nikola, Tselinnitsa, Taganrog, Bezenchukskaya 210, Bezenchukskaya krepost, Bezenchukskaya jubileynaya, Bezenchukskaya 205, Tessadur. Sources of resistance are intraspecific variability and introgression of genetic material from other species, primarily T. dicoccum Schuebl., T. timopheevii Zhuk., T. aestivum L. In particular, the lines NT-7, NT-10, and NT-12 that are highly resistant to stem rust, obtained by selection in F3BC1 Shortandinskaya 71/Orenburgskaya 2//T. timopheevii k-38555/3/ Shortandinskaya 71 are used as initial material (Kozlovskaya et al., 1990). The resistance of the obtained lines is well inherited and controlled by groups of 3–4 genes with the manifestation of complete dominance, incomplete dominance, recessive control of resistance (Khlebova, Barysheva, 2016).

In Western Siberia, on the genetic material of soft wheat from CIMMYT on a natural infectious background during 2018–2019, a high resistance to the stem rust population of the genes Sr23, Sr31, Sr38, Sr39, Sr40 and combinations of the genes Sr6, Sr24, Sr36 and 1RS-Am, Sr21, Sr31 (Evdokimov et al., 2022). Therefore, there are no corresponding virulence alleles in the stem rust population.

Also in the same studies, the high resistance of the cultivars Triada, Omsky coral, Odisseo was determined. The average susceptibility was noted in Omsky izumrud and Luch 25. The vast majority of commercial varieties from Russia and Kazakhstan are very sensitive to race Ug99 which was determined in tests against a natural background in Kenya (Shamanin et al., 2016). At the same time, it was possible to identify genotypes resistant to Ug99 among the breeding material of which three samples (Gordeiforme 178-05-02, Gordeiforme 05-42-12, and Triada) were resistant or moderately resistant in Kazakhstan and Russia. Stem rust reactions in Kenya and Kazakhstan were similar. In Western Siberia (Omsk, Barnaul, 2017–2018), the degree of damage was higher. Scientists of the Global Rust Reference Centre found that P. graminis races from Omsk have unusual virulence patterns compared to Ug99 and races from other regions (Hovmøller et al., 2017).

In the Eurasian regions with a hot climate where spring durum wheat is mainly cultivated, epiphytotics of powdery mildew (Blumeria graminis) take place. This can be explained by a short incubation period which at an average daily temperature of 20 to 24 °C ranges from 2.8–3.5 days (Fissyura et al., 1987) that in the presence of an infection ensures its rapid spread. Powdery mildew epiphytoties negatively affect grain quality reducing the content of protein, gluten, weight and grain size (Dolgalev, Tikhonov, 2005). In this regard, resistant cultivars are included in the Register of Russia and Kazakhstan, including steppe regions with a hot climate.

18 spring durum wheat cultivars of 61 ones included in the State Register of Russia show high resistance (R, RMR) to powdery mildew. Analysis of the breeding records of these cultivars shows various sources of resistance, including those based on the genetic variability of T. durum, T. dicoccum, T. timopheevii. In particular, the cultivars Bezenchukskaya krepost and Taganrog which are resistant to powdery mildew carry a translocation from T. timopheevii on the 6B chromosome range of microsatellite markers Xgwm518 and Xgwm1076 (Malchikov et al., 2015). Lines 1438D-13 (resistance donors – T. timopheevii, T. durum), 1389DA-1, 1477D-4 (resistance donors – T. durum, T. dicoccum) with immunity to powdery mildew were obtained at the Samara Research Institute of Agriculture (Malchikov et al., 2015).

A grain with a “black germ” is the main reason for the presence of spex (dark inclusions) in the grains that reduces the colour and nutritional quality of pasta in general (Vasilchuk, 2001). The black germ appears as a result of grain infection with pathogens B. sorokiniana (Sacc.) Shoemaker, Alternaria tenuis (Fr.), A. tritici (Pers.) during the filling period (Conner, 1987).

The assumption that large-grain cultivars are more susceptible was not confirmed – in the process of breeding, resistant cultivars with different grain weights were obtained. Under the conditions of the Altai Territory, the following cultivars are classified as resistant: Salut Altaya, Pamyati Yanchenko, Altaisky yantar, Solnechnaya 573, Angel, Omsky izumrud, 1480d-2, Luch 25, Kharkovskaya 46, Donskaya elegiya, Orenburgskaya 10 (Barysheva et al., 2016). The Samara Research Institute of Agriculture (Malchikov et al., 2022) also identified resistant cultivars: Kharkovskaya 46, Bezenchukskaya 139, Bezenchukskaya 182, Marina, Taganrog, 1963D-71, 2021D-1, Gordeiforme 910, Gordeiforme 08-25-2, Gordeiforme 08-107-5, Melyana. Highly resistant durum wheat genotypes from Italy – ISD19, ISD20, ISD22, Аchille, Greсalle, Odisseo and Austria – Duroflaus and Duromax were proposed as initial material.

In Eurasia, significant damage to the yield and quality of durum wheat grain is caused by pests. The leech, bread beetles, thrips, turtle bug have a focal distribution pattern with a rare manifestation of signs of epizootic in some regions. Reducing the harmfulness of these pests is provided by agrotechnical methods. Selection for resistance to Hessian flies, Swedish flies, and the grain sawfly is quite effective (Vyushkov, 2004).

Hessian fly resistance is controlled by a block of dominant Н1 – Н24 genes and several recessive genes (McIntosh et al., 2013). The varietal population of durum wheat in Russia has a sufficient concentration of these genes. The genetics of resistance to the Swedish fly is less studied, but modern cultivars of durum wheat show relative resistance to the pest – damage rarely reaches 11–15 % (Blagonadezhdina, 1968). Kharkovskaya 46 and Bezenchukskaya 139 are evaluated as genetic donors of resistance to the Swedish fly (Vyushkov, 2004).

The control system of breeding material in seed-plots and statistical analysis of varietal variability makes it possible to create slightly damaged cultivars. Resistance to the grain sawfly ensures that the stem is solidness with parenchymal tissue. The high heritability of the trait and the control of the system of dominant genes in interaction with genes inhibitors and anti-inhibitors of stem core formation (Malchikov, Myasnikova, 2008) determine the efficiency of selection of cultivars with a fully or partially completed culm. The absence of negative effects of the genes that control straw solidness on the production process and grain quality allows them to be widely used in breeding for all regions (Malchikov, Myasnikova, 2008). The use of cultivars with completed straw is most effective in the southern regions where the grain sawfly causes maximum damage to the yield and grain quality (Kryuchkov, 2006). Among the cultivars of durum wheat approved for use in 2022, 4 have a fully solidness culm, 36 – medium and 21 cultivars – hollow straw (State Registers of Selection Achievements…, 1993–2022).

## Selection for drought tolerance

N.I. Vavilov (1935) attached great importance to the creation of drought-resistant cultivars. The property itself of drought resistance was considered by him as extremely dynamic, depending on time, duration of stress and the period of plant ontogenesis. In his opinion, drought resistance is not a specific trait permanently and invariably inherent in one or another cultivar (Vavilov, 1935). Nevertheless, P.N. Konstantinov (1923) singled out the most significant features which determine drought resistance – the development of the root system and precocity. Subsequently, P.A. Genkel (1982) identified the following factors of drought resistance: 1) resistance of the cytoplasm to dehydration and overheating; 2) rhythm of development; 3) development of the root system; 4) potential productivity.

According to V.A. Kumakov (1985), the weak cytoplasm resistance reduces agronomic drought resistance. Physiological (cytoplasmic, cellular) resistance is considered by breeders as the basis for root growth in soil with low humidity (Kumakov, 1985; Vasilchuk, 2001). Stronger inhibition by drought of cell division in the zones of durum wheat apical meristems than in soft wheat is the main reason for the decrease in its fertility and tillering (Kumakov, 1985).

Selection of spring durum wheat in Russia and the CIS countries for a long period (50–112 years) is carried out in the steppe regions under conditions of significant drought pressure. During this time, tens of thousands of hybrid combinations and millions of breeding lines have gone through cycles of natural and artificial selection. Modern cultivars in drought conditions exceed grain landraces by 2 times in productivity, the first breeding cultivars – by 1.5–1.8 times (Gaponov et al., 2017).

Drought resistance of cultivars is determined by the degree of adaptation to the regional dynamics of growing season meteorological factors. Krasnokutskaya Breeding Station selects the most early-ripening cultivars in the Commonwealth of Independent States (CIS). This is due to the high probability of developing a spring-summer drought with high temperatures in this zone. Early maturing cultivars which form an acceptable yield due to autumn-winter precipitation are an expedient agroecotype here. In this region, early maturation must be combined with a strong root system (Konstantinov, 1923) which due to the reduction in the total growth period is a difficult task for breeders. It is necessary to take into account the decrease in the root system size caused by the negative effect of the dominant Vrn-A1 gene allele (Smirnova, Pshenichnikova, 2021).

The cultivars bred by the Research Institute of Agriculture of the South-East also mainly belong to the early-ripening biotype. Like the Krasnokut ones, they have a high field drought resistance and are well adapted to the conditions of the Lower Volga region. Cultivars Krasnokutka 13, Nikolasha and Saratovskaya zolotistaya should be considered a significant success in breeding drought-resistant cultivars of these institutions. The first two combine this property with precocity. The third one has a high heat resistance of the ear and has a good cultivar-forming ability in the Middle Volga region and in the Urals (Malchikov, Myasnikova, 2015). Under drought conditions, when studying a set of contrasting cultivars of the Volga region by the method of principal components, a cluster of traits was identified that is closely and positively related to productivity: the number of grains per spike, the nitrogen harvesting index, Harvest index, the growth function of the spike during flowering, the removal of nitrogen and phosphorus during the period from flowering to maturation, the leaf area of the main shoot in tillering (Malchikov, Myasnikova, 2015).

The positive relationship between the removal of macronutrients and, first of all, phosphorus, with the yield during the drought can be interpreted as a result of more vigorous growth of the root system (formation of root hairs) and its activity in drought-resistant varieties (Reynolds et al., 2012). Based on the results of many years of research, drought-resistant cultivars have been identified in Samara Research Institute of Agriculture which are widely used in breeding as initial material: Pamyati Chekhovicha, Bezenchukskaya 205, Bezenchukskaya zolotistaya, Marina, Bezenchukskaya 207, 653d-53, 1368d-18, 2034d-41 (Samara Research Institute of Agriculture), k-16441 (Saada – Morocco), D2017/Karasau//D2043 (Research Institute of Agriculture of the South-East). In the KASIB system, 34 drought-resistant samples were identified out of 154 genotypes during 2000–2015; some of them were later released in arid regions (Evdokimov et al., 2017).

In the Rostov Agricultural Research Centre, successful breeding for drought resistance is carried out using chemical mutagenesis combined with hybridization (Kadushkina et al., 2016). The cultivars of this institution, Donskaya elegiya, Melodiya Dona and Donella M, have high drought resistance in the southern region and in the Volga region. The high drought resistance of cultivars of the National Grain Centre named after P.P. Lukyanenko – Yasenka, Yadritsa and Yarina, and Altai Scientific Centre of Agrobiotechnologies – ATP Prima, ATP Partner, Shukshinka was manifested in the Middle Volga region in the system of ecological testing. The Orenburg Research Institute of Agriculture found that drought-resistant durum wheat cultivars are an effective component of conservation technologies that compensate their negative effects associated in some cases with soil compaction and reduced fertility (Besaliev, Kryuchkov, 2014).

M. Reynolds (2012) with co-authors identified as the main components of the complex property “drought resistance”: CTV/CTG – leaf surface temperature at the stages of vegetative growth and grain filling; GC – soil cover during the formation of the crop; ANT – the number of days before flowering; CAR – concentration of carotenoids in leaves; TE – transpiration efficiency based on carbon isotope discrimination; WSC – concentration of sugars in the stem immediately after flowering; HI – Harvest index.

Parameters CTV/CTG, TE are largely determined by the depth of penetration and activity of the root system, xylem diameter and stomatal density. This complex allows you to extract water from the soil, maintain normal transpiration and photosynthesis. The genes TaMOR, TaERs have been described on soft wheat. The first one is a transcription factor activated by auxin affects the growth and number of roots. The second one (includes TaER-1, TaER-2) increases the density of stomata and their conductivity, reduces the size of epidermal cells and TE that has a positive effect on photosynthesis and the accumulation of plant biomass in drought conditions

The study of genes and transcription factors which are orthologous to the structures of other species (arabidopsis, rice, maize) will make it possible to understand the mechanisms of drought and heat resistance in wheat (Kulkarni et al., 2017). V.A. Dragavtsev et al. (2017) suggested using approaches based on the ecological-genetic theory of the organization of quantitative traits in the selection of drought-resistant cultivars. According to their ideas, drought resistance is included in the genetic and physiological system of adaptability and contains 22 component traits. The acceleration of breeding for drought resistance involves the identification of the physiological characteristics of cultivars created in breeding centers located in regions that differ in types of dominant droughts and the dynamics of meteorological factors in plant ontogenesis. Knowledge of specific physiological, epigenetic, biochemical components of drought resistance in the future will make it possible not only to obtain transgressive forms in the process of recombinant breeding, but also to mark the corresponding QTLs for the formation of marker-associated breeding technology. For each agroecological zone, it is necessary to determine the parameters of morphotypes that are complementary to the main types of drought.

## Breeding of short-stemmed cultivars

The breeding of short stem varieties of spring durum wheat carried out in the former USSR in the 1970s, was not successful (Vyushkov, 2004; Golik V.S., Golik O.V., 2008). The main reason for the failure was the low adaptability of the short-stemmed donors from Mexico, Chile, Italy, Australia, and Canada. Resistance to lodging in arid zones is expected to be improved by increasing stem strength. (Vasilchuk, 2001). However, the benefit of reducing plant height is not limited to increased resistance to lodging. Low-growing cultivars have a high grain yield from total biomass and productivity potential which is confirmed by the history of wheat breeding in the XX century in many countries.

In Russia, plant height reduction genes are used in the selection of soft and durum winter wheat. Much more difficult is their use in the breeding of spring wheat that is more severely affected by drought than winter wheat. The creation of short-stemmed analogues of Russian cultivars and the gene study on the material of isogenic lines turned out to be promising (Gurkin, 1984; Vyushkov, 2004). By expression at plant height in the Volga region, the studied genes are distributed in the following order: Rht 14 >RhtB1b >RhtAz >RhtAhn. The Rht 14 gene was recognized as unpromising because of strong negative effects on adaptability. The RhtB1b gene reduced the height of plants by 40 %, increased the grain size of the ear by 17 %, the total tillering by 15 %, Harvest index by 11 %, reduced the weight of 1000 grains by 9 %. The RhtAz and RhtAhn genes reduced plant height by 17 and 12 %, respectively. The effects of the latter genes on productivity elements were insignificant (Gurkin, 1984; Vyushkov, 2004).

A.A. Alderov (2001) at the Dagestan Experimental Station of All-Russian Institute of Plant Genetic Resources (VIR) introgressed into T. durum the genes controlling short stature from the diploid species T. sinskajae (SIS2) from the hexaploid species T. aestivum – Tom Pouce (Rht 3) and from T. dicoccum – k-25459 (rhtx 1, rhtx 2). The use of these genes was recommended in the North Caucasus. In the Samara Research Institute of Agriculture, on the basis of an analogue of Kharkovskaya 46 (RhtAhn gene), a droughtresistant cultivar Pamyati Chekhovicha was obtained which transferred its properties, including the gene for reducing plant height, to cultivars: Bezenchukskaya 210, Bezenchukskaya zolotistaya, 1368D-18, Bezenchuksky podarok (Samara Research Institute of Agriculture), Shukshinka, ATP Prima (Federal Altai Scientific Centre for Agrobiotechnology), Kremen (Federal Scientific Center for Biological Systems and Agrotechnologies of the RAS). The RhtB1b gene was introduced into the commercial varieties Bezenchukskaya 209 and Triada.

In the same period, short-stemmed foreign cultivars Sea Nillo, Sea Atlanta, Tessadour were released. The cultivars Bourbon and Nikola created on genetic material from Italy have a shortness phenotype. The cultivars Bezenchukskaya 209 and Triada have a sufficient level of drought resistance and can be used with intensive tillage technologies in the steppe regions of Russia with an arid climate. Thus, purposeful and long-term work with carriers of plant height reduction genes made it possible to create competitive low-growing/medium-sized varieties and move on to the formation of new morphophysiological types of spring durum wheat in various regions of Russia.

## Breeding for grain quality traits

At the end of the XIX century and the beginning of the XX century, the quality of Russian durum wheat on the European market was beyond competition (Chekhovich, 1924). At that time, the main criteria for grain quality were: vitreousness, protein content, completion, grain weight and colour. In 1929 in Saratov (Research Institute of Agriculture of the South-East) A.I. Marushev (1968) began to study in addition to these features in a specialized laboratory: baking properties, strength of flour (gluten), breaking strength macaroni, pasta colour, digestibility, loss of solids when cooking pasta. These parameters formed the basis for evaluating varieties in other breeding centres, in the state commission and formed the basis of GOST for durum wheat quality classification. At present, classiness is determined by vitreousness, grain nature, the presence of grains with a black germ, the quantity and quality of gluten (using the GDI-1-Gluten deformation index device), and the falling number.

In the late 1980s, N.S. Vasilchuk (2001) proposed to evaluate the quality of durum wheat varieties by groups of traits: 1) determined on the grain (glassiness, weight of 1000 grains, nature, colour, ash content, falling number, protein content); 2) determined on meal: the number of spices, colour, content of yellow pigments and activity of oxidizing enzymes; 3) rheological properties of the dough – SDS sedimentation, parameters determined on a mixograph, farinograph, alveograph, glutomatic, glutograph, electrophoresis of gliadin and gutenin blocks, DNA markers); 4) culinary properties of pasta (colour, strength of dry and cooked products, boilability, amount of solids in cooking water).

The most difficult for breeding are signs that negatively correlate with yield, weight of 1000 grains and grain volume weight. They include the protein content in the grain and the amount of gluten. In the process of long-term breeding, these contradictions have become aggravated. Attempts have been made to overcome or significantly reduce the effects of this negative dependence. The discovery of the wild emmer wheat T. dicoccoides (FA-15-3) with large grains and high protein content in Israel made it possible to mark the corresponding locus QGpc.ndsu- 6Bb on the short arm of the 6B chromosome in the region Xabg387-6B and Xmwg79-6B where 11 markers are located (Joppa et al., 1997). This made it possible to identify high Gpc protein loci in collections of wild species, landraces, and breeding cultivars. The presence of translocation in the group of modern cultivars shows that joint selection for protein content and productivity is possible. In Russia, many cultivars of durum wheat have Kharkovskaya 46 in their breeding record obtained with the participation of the T. dicoccum and its sister line Kharkov- skaya 51 and other cultivars whose ancestors are T. dicoccum, T. timopheevii. That suggests a certain probability of their having Gpc genes

The study of 38 durum wheat cultivars created in different ecological and geographical zones (Kharkov, Rostov, Saratov, Bezenchuk, Omsk, Altai) for three years at four points (Bezenchuk, Kurgan, Barnaul, Aktyubinsk) made it possible to determine the following evolutionary grain yield and protein content trends: 1) an increase in the protein content in the grain while maintaining the intensity and adaptability of the production process at the level of the previous breeding stages (cultivar Solnechnaya 573); 2) a significant and stable improvement in yield properties during the breeding process is not accompanied by a decrease in the protein content in the grain (cultivar Bezenchukskaya krepost); 3) a significant and stable improvement in yield properties during the selection process is accompanied by a significant decrease in the protein content in grain (cultivars Bezenchukskaya niva, Bezenchukskaya 210) (Myasnikova et al., 2019).

In addition to protein and gluten content, significant efforts by breeders have been directed towards improving the gluten quality. A.I. Marushev (1968) established a connection between the strength and cooking properties of pasta and the baking properties and strength of flour. The strength of the flour did not always determine the baking qualities of durum wheat, but it was also more closely related to the strength and cooking properties of pasta. The possibility of combining good pasta and baking qualities in durum wheat was also established. Gordeiforme 432, Melyanopus 26, Saratovskaya 34 cultivars were assigned to such ones at the Research Institute of Agriculture of the South-East. Later, V.S. Golik and O.V. Golik (2008) showed the possibility of creating durum wheat cultivars with good baking properties. Canadian breeders came to a similar conclusion where the expediency of breeding durum wheat cultivars of dual use was substantiated

The discovery of R. Damidaux et al. (1978) two components of γ-gliadin designated γ-42 and γ-45 which were found to be markers of weak and strong gluten, respectively, was important for breeding durum wheat cultivars with high gluten quality. The strong gluten of the γ-45 genotypes (allele) is now known to be functionally mediated by a specific group of low molecular weight (LMW-GS) glutenin subunits designated LMW-2, closely linked to Gli-B1dc (γ-45) (Pogna et al., 1988). GliB1dc (γ-45)/ LMW-2 white spike genotypes were identified early on. Subsequently, A.M. Kudryavtsev (1994) identified two biotypes, γ-45 and γ-42, in the Kharkovskaya 3 cultivar with red ear. The LMW-2 durum wheat genotypes have a wide range of gluten strengths, but they almost always outperform the LMW-1 (γ-gliadin 42) genotypes in baking quality (Kosmolak et al., 1980). There is no evidence that stronger LMW-2 genotypes are better in pasta quality than weaker LMW-1 genotypes (Marchylo et al., 2001). However, strong gluten with its raw content of 28.0–35.0 % has high technological properties in the manufacture of pasta as it gives a dense, viscous dough, well molded, elastic, not wrinkled, not sticky when extruding pasta (Marushev, 1968; Savitskaya et al., 1980).

It has now been established that the quality of durum wheat gluten is determined mainly by five loci, two of them, Glu-A1 and Glu-B1, control the synthesis of high molecular weight glutenins (HMW-GS), three, Glu-A3, Glu-B2, Glu-B3, control the low molecular weight glutenins (LMW-GS). In the Glu-B1 locus, a positive effect on the quality of gluten (according to the SDS test) was found for alleles b (7+8), d (6+8), z (7+15), ch (7+12), in the Glu-B3 for allele a (2+4+15+19), in the Glu-A3 locus – for alleles a (6), c (6+10), d (6+11), e (11). The combination of different alleles at the loci forms more than 40 haplotypes (Ronchallo et al., 2021). Obviously, the study of new varietal collections will make it possible to identify new alleles and determine their effect on the quality of gluten. In Russia, the polymorphism of glyadin-coding loci was studied on the material of collections of historical and modern varieties. In 46.0 % of the cultivars included in the Russian register for 2014, the block Gli-B1dc (γ-45)/LMW-2 was found, which implies a high efficiency of breeding high-quality varieties.

Judging by the genealogy of cultivars approved for use in Russia in 2022, it can be assumed that the number of cultivars with high gluten quality (presumably having LMW-2) has increased. Since the end of the 1980s, selection for the quality of gluten has been carried out according to the parameters of GDI (gluten deformation index), SDS sedimentation, mixograph, farinograph. In the Research Institute of Agriculture of the South-East, they improved the assessment of cultivars according to SDS sedimentation (microsedimentation), proposed to expand the 8-point scale of the mixogram at the beginning to 9-point scale (Vasilchuk, 2001) and then to 10-point scale (Gaponov et al., 2020) which was caused by the creation of high-quality cultivars that, under local conditions, form heavy-duty gluten that does not fit into the parameters of the 8-point scale. Currently, the quality of gluten is assessed additionally using glutomatic and glutograf devices (Gaponov et al., 2020). LLC “Agroliga Center for Plant Breeding” and Samara Research Institute of Agriculture using the biochemical marker Glu-B1 (7+8) and molecular markers of the microsatellite group SSR – Single sequence Repeat (Barc148) and SNP – Single Nucleotid Polimorphism (BM140362) linked to the genes of high-molecular glutenins on the 1A chromosome created two cultivars – Taganrog and Alazar (Shevchenko et al., 2019).

In the future, the widespread use of marker-associated breeding technology for these traits based on extensive studies on the phenotyping and genotyping of the gluten quality of collections and commercial durum wheat cultivars adapted to environmental conditions in various agroecological zones of Russia is expected.

Significant progress has been made in breeding for the content of yellow pigments in the grain. This trait is quantitative and under the control of genes with strong additive effects. The corresponding QTLs are distributed over all chromosomes of the durum wheat genome. The trait variation is 60 % determined by two QTLs located on chromosomes 7AL and 7BL (Elouafi et al., 2001; N’Diaye et al., 2017). The first breeding cultivars (created in the 1920–1940s) – Gordeiforme 432, Melyanopus 69, Gordeiforme 189, Gordeiforme 675, Melyanopus 26, accumulate 3.6–5.0 ppm of yellow pigments in the grain. The same level or slightly higher had cultivars of 1960–1980s Kharkovskaya 46, Bezenchukskaya 105, Bezenchukskaya 139. Cultivars Svetlana (1987) and Saratovskaya zolotistaya (1993) accumulated in grain 6–6.5 ppm and 7–7.5 ppm, respectively, that exceeds the level of the first grade of scientific breeding – Gordeiforme 432 by 25–55 %.

Among the cultivars released since 2016, Bezenchukskaya zolotistaya (8.5–9.0 ppm), Bezenchukskaya krepost and Tamara (7.5–8.5 ppm) stand out noticeably. All genotypes of foreign origin were inferior to these cultivars in terms of the trait size when studied in the breeding centres of Russia. The concentration of yellow pigments in the grain of these cultivars exceeds Gordeiforme 432 by 65–85 %. The cultivars Bezenchukskaya zolotistaya, Bezenchukskaya krepost, Bezenchukskaya 210 and Saratovskaya zolotistaya with the optimal combination of the amount of pigments, adaptability, stability and responsiveness of their accumulation in grain were identified. These genotypes are most appropriate to use for the formation of populations and the creation of recombinant inbred lines followed by mapping of the corresponding QTLs and the creation of marker-associated selection technology (Malchikov, Myasnikova, 2020).

Thus, most modern cultivars of spring durum wheat surpass the cultivars of the first breeding stages in terms of the content of yellow pigments in the grain, the rheological properties of the dough and the culinary quality of pasta – primarily the colour and the strength of boiled pasta.

## Conclusion

The history of durum wheat growing in Russia and in the former USSR covers many centuries. It is associated with the spread of agriculture in the steppe regions of the Kuban, the Volga region, Siberia and Kazakhstan. At present, about 0.8 million hectares are sown annually in Russia. That is much less than during the period of the planned economy. Scientific breeding of durum wheat has been carried out since 1909. Its yield trend results, depending on the period and region, are 0.26–1.0 % per year, quite comparable with similar results in other countries with a long history of durum wheat breeding. The genetic core of modern varieties was formed on the basis of local varieties of durum wheat, their hybridization with T. aestivum L. and T. dicoccum Shuebl. The attraction of source material from other countries that has recently increased has had a positive effect.

Despite a significant number of durum wheat breeding laboratories in Russia and a variety of source material, there is a decrease in diversity for alleles of gliadin-coding loci and erosion of original Russian ancestors in modern varieties. Significant advances have been made in breeding for resistance to dust brand (currently 40 % of commercial varieties are resistant) and the most harmful pathogens causing leaf spot in Eurasia (Stagonospora nodorum Berk., Septoria tritici (Roeb. et Desm.), Bipolaris sorokiniana (Sacc.) Shoemaker, Pyrenophora tritici-repentis, Fusarium sp.). Varieties immune to leaf rust (Рuccinia triticina) (immunity type 0-1) along with field resistance created in Russia and Kazakhstan are recommended for regions with an increased level of annual precipitation. According to the State Commission for Testing and Protection of Breeding Achievements, 14 varieties out of 63 ones included in the Register of Russia showed resistance to P. graminis f. sp. tritici from medium to high against a natural infectious background.

Breeding of the cultivars resistant to B. graminis (DC.) f. sp. tritici Em. Marchal. is being carried out effectively – 30 % of the cultivars show high resistance to this pathogen and pathogens that cause blackening of the corcule and the endosperm. High performance was achieved in breeding for resistance to Hessian, Swedish flies and grain sawfly. The varietal population of durum wheat in Russia has a sufficient concentration of dominant (Н1 – Н24) and recessive genes that determine resistance to the Hessian fly. Most Russian durum wheat cultivars show relative resistance to Swedish fly – damage rarely reaches 11–15 %.

Selection for drought resistance is regional in nature. It means that drought-resistant biotypes are formed depending on the amount of precipitation, temperature and their dynamics in the regions. Drought-resistant cultivars with the Rht Ahn plant height reduction gene (Bezenchukskaya zolotistaya, Bezenchukskaya 210, Bezenchuksky podarok, Shukshinka, ATP Prima) and cultivars Bezenchukskaya 209 and Triada carrying the RhtB1b gene adapted to the steppe arid zones were created.

Improvement of grain quality in addition to physical properties is carried out in terms of protein content, amount of yellow pigments and gluten quality. A pair of traits “protein (gluten) content – yield” in the breeding process evolved in the following degree of conjugation: 1) an increase in protein concentration while maintaining yield; 2) decrease in protein concentration with a significant increase in yield; 3) increase in yield while maintaining protein concentration. The concentration of yellow pigments in the grain of modern cultivars exceeds the indicator of the first breeding cultivar Gordeiforme 432 by 65–85 % and reached 8–9 ppm in a number of cultivars. A significant improvement in the quality of gluten during the selection process occurred in terms of GDI (gluten deformation index), sedimentation (SDS), mixograph, farinograph parameters, and gluten index. According to the results of electrophoresis of the gliadin fraction of storage proteins, the presence of a low molecular weight component of glutenin of the second type (LMW-2) functionally associated with the formation of high-quality gluten was revealed in 50 % of commercial cultivars

In the short and medium term, classical breeding approaches will continue to play an important role in the durum wheat improvement. Advances in DNA sequencing and other technologies such as bioinformatics, statistics, etc. can help breeders improve the efficiency and speed of the breeding process. Finally, the use of new molecular biology technologies is essential, but their applica-tion must be combined with reliable and extensive field testing.

## Conflict of interest

The authors declare no conflict of interest.
